# Glucose concentration modulates bovine intervertebral disc response under physiological loading

**DOI:** 10.3389/fbioe.2026.1751565

**Published:** 2026-03-26

**Authors:** D. Zuncheddu, M. Muerner, K. Klavins, M. J. Stoddart, L. B. Creemers, S. Grad

**Affiliations:** 1 AO Research Institute Davos, Davos, Switzerland; 2 Department of Anesthesiology, MUMC/Mental Health and Neuroscience Research Institute, Division Translational Neuroscience Maastricht University, Maastricht, Netherlands; 3 ETH Zürich, Zürich, Switzerland; 4 Institute of Biomaterials and Bioengineering, Faculty of Natural Sciences and Technology, Riga Technical University, Riga, Latvia; 5 Baltic Biomaterials Centre of Excellence, Headquarters at Riga Technical University, Riga, Latvia

**Keywords:** *ex vivo*, glucose metabolism, intervertebral disc, mechanical loading, organ culture, sex differences

## Abstract

**Introduction:**

*Ex vivo* organ culture is a widely accepted model in intervertebral disc (IVD) research, providing valuable insights into pathology and potential new treatments. Many *ex vivo* culture protocols utilize high concentrations of glucose (25 mM), which do not reflect IVD physiology *in vivo*. In this study, we investigated effects of varying glucose concentrations on bovine IVDs in a bioreactor-loaded organ culture. In addition, given the growing body of literature showing differences in male vs*.* female in connective tissue diseases, we verified whether sex affected the responses.

**Methods:**

Whole IVDs were cultured under physiological loading (0.02–0.20 MPa, 0.2 Hz, for 2 h per day) with different glucose supplements (25 mM, 11 mM, and 5.5 mM) for 1 week. The effects of glucose supplementation on IVD cell viability, metabolism, disc height, glycosaminoglycan (GAG) release, and the expression of key anabolic and catabolic markers were analyzed.

**Results:**

Decreasing glucose concentration had a dramatic influence on the viability of bovine IVD cells. Under 25 mM glucose, high viability was maintained after 7 days, reaching approximately 80% in the annulus fibrosus (AF) and 72% in the nucleus pulposus (NP). At the lower glucose concentrations (11 and 5.5 mM), cell viability decreased significantly to approximately 31% and 7.5% in the inner AF, and 27% and 11% in the NP. Metabolomic analysis showed reduced levels of metabolites related to β-oxidation, antioxidant protection, and osmoregulation from day 1 to day 7, suggesting an adaptive response to the *ex vivo* environment. Although no significant sex differences were observed in cell viability, the expression of apoptotic markers in NP was significantly higher in the male cohort for the 5 mM glucose groups. Additionally, anabolic and glucose transporter markers were significantly elevated in the male compared to the female cohort in a region- and glucose-dependent manner, underscoring the sex-dependent responses of disc cells to varying glucose concentrations.

**Conclusion:**

This study enhances our understanding of bovine disc cell metabolism during organ culturing. Furthermore, the findings emphasize the need to optimize nutrient and loading conditions in *ex vivo* IVD models to more closely mimic *in vivo* physiology.

## 1 Introduction

Intervertebral disc degeneration (IDD) is a complex, multifactorial degenerative process, impacting the structural integrity and biochemical composition of the intervertebral discs (IVD) that interconnect the adjacent vertebrae. While often attributed to the natural aging process, several extrinsic and intrinsic factors can expedite IDD, including genetic predisposition, obesity, and metabolic disorders (Francisco et al., 2022; Fine et al., 2023; Yi et al., 2023; Li et al., 2024). The degeneration occurs through a series of structural modifications in the extracellular matrix (ECM), leading to a diminished disc height and an increased risk of nucleus pulposus (NP) herniation. These alterations can severely impair spinal function and have significant implications for clinical outcomes, including chronic pain and disability. The accelerating prevalence of IDD due to aging, lifestyle factors and metabolic conditions is a growing concern for public health (Cannata et al., 2020). A comprehensive understanding of the underlying mechanisms and risk factors associated with IDD is imperative for developing targeted prevention and treatment strategies, ultimately enhancing patient care and quality of life.

The IVD is the largest avascular structure in the human body, relying on the diffusion of nutrients from the cartilage endplates (CEPs) and outer annulus fibrosus (AF) to maintain the viability of its cellular components (Ashinsky et al., 2020). The AF and the NP therefore are exposed to distinct nutritional conditions and changes in nutrient supply (De Geer, 2018). Glucose has been identified as the primary nutrient utilized by disc cells for energy production and ECM maintenance (Yin et al., 2020). Given the relative lack of oxygen in the IVD, energy generation is assumed to mainly occur through glycolysis, leading to a characteristically low pH in the IVD due to the associated lactate production. However, it is not clear to what extent other sources of energy are used.

*Ex vivo* IVD organ culture has become a well-accepted model in the field of IVD research, due to its capacity to maintain the complex physiological environment of the IVD, providing precise control over various experimental parameters, offering significant ethical and practical advantages by reducing animal use and costs, and enabling detailed mechanistic studies through advanced imaging and controlled biochemical environments (Tang et al., 2022). Thereby organ cultures can provide valuable information on pathology, and they can be used in the development of new treatments.

Although direct measurements of glucose in humans are lacking, the physiological glucose concentration within the IVD is estimated to be approximately 1–4 mM, based on *in silico* modelling of nutrient transport (Soukane et al., 2007; McDonnell and Buckley, 2022). Often, *ex vivo* culture protocols employ high concentrations of glucose (25 mM), which do not reflect the IVD physiology *in vivo*. Research on IVD cell and tissue cultures has explored various glucose concentrations to optimize the culture conditions of *ex vivo* models. Limited glucose (11 mM) significantly reduced cell viability in whole ovine IVDs compared to sufficient (25 mM) glucose (Jünger et al., 2009), and high-frequency loading had an additive effect on cell death (Illien-Junger et al., 2010). For human IVDs cultured with CEPs, both low (5.5 mM) and high (25 mM) glucose concentrations could maintain cell viability, while removal of the CEP caused cell death due to rapid swelling and deformation (Gawri et al., 2011). Bovine NP cells in explant culture were shown to survive in harsh conditions, such as low glucose (5.5 mM), while dynamic loading increased the metabolic activity of the NP cells (Salzer et al., 2022). In 3D bovine NP cell cultures, glucose omission led to increased cluster size, apoptosis, and senescence (Stephan et al., 2011). Moreover, computational modelling studies have evaluated that a glucose concentration of 25 mM for *ex vivo* culturing of bovine IVDs resulted in a supraphysiological level of glucose (8–12 mM) in the centre of the disc (McDonnell and Buckley, 2021). These findings highlight the importance of glucose concentration in IVD culture, with optimal levels varying depending on cell type, species, and culture system. Nevertheless, the medium glucose concentration that maintains the viability of bioreactor-loaded bovine IVD whole organ cultures has not been evaluated yet.

Biological sex has emerged as a significant factor influencing the varying susceptibility to IDD, with epidemiological data indicating a higher prevalence in women compared to men (Teraguchi et al., 2014; Parenteau et al., 2021). Estrogens play a role in modulating metabolic functions such as vascular perfusion and nutrient diffusion, and their deficiency may worsen degenerative processes in the avascular IVD (Shelby et al., 2023). Despite this demonstrated and suspected impact, biological sex has not been considered in *ex vivo* IVD studies to date (Zuncheddu et al., 2025).

Therefore, this study aimed to: i) determine the level of glucose that maintains cell viability and phenotype in young bovine caudal IVDs in bioreactor-loaded organ culture; and ii) as a secondary and exploratory objective, investigate potential differences between female and male donors. By incorporating sex-disaggregated analysis in a bovine *ex vivo* model, this study provides novel insight into potential sex-dependent adaptation of disc cells under different concentrations of glucose. Refining *ex vivo* culture protocols is fundamental for creating suitable models for IDD and assessing potential regenerative treatments.

## 2 Methods

### 2.1 Dissection, culture, and loading of bovine IVDs

Eleven freshly slaughtered calve tails (6 F/5 M; age 8.81 ± 2.95 months) obtained from a local abattoir were dissected, and five IVDs (3 for culture +2 day 0 controls) per tail were isolated as previously described (Saravi et al., 2021). Briefly, tails were rinsed with Lifo-Scrub® (B.Braun, Germany) and immersed in 1% betadine solution (Mundipharma, Germany) for 10 min. Soft tissue was removed, and bony processes were cut using bone pliers. Two parallel cuts were made through the growth plates at both ends of the IVDs using an Exakt 300 Band Saw (Exakt, Germany). IVDs were cleaned using a Pulsavac® (Zimmer Biomet, Switzerland) wound debridement system for 30 s, washed in a 10% penicillin-streptomycin (Pen-Strep; Gibco, Life Technologies, United States of America) solution for 12 min, followed by a 2-min rinse in a 1% Pen-Strep solution. All IVDs were randomly assigned to one of three experimental groups.

After overnight free swelling culture, whole IVDs were cultured under physiological conditions of uniaxial loading (0.02–0.20 MPa, 0.2 Hz, 2 h/day) in a bioreactor, and with varying glucose supplementation for 1 week (from day 1 until day 7). The bioreactor system applies controlled uniaxial cyclic compression to whole bovine IVDs, and operates under force control within a standard incubator, as previously described (Li et al., 2020). Discs are placed in custom-made chambers and loaded *via* two porous plates, which allow nutrient exchange while maintaining stable axial alignment through a constant compressive preload.

IVDs were cultured in DMEM medium containing 4.5 g/L (25 mM), 2 g/L (11 mM) or 1 g/L (5.5 mM) of glucose and supplied with 2% fetal calf serum, 1% ITS + Premix (Discovery Labware, Inc., Bedford, United States), 50 μg/mL ascorbate-2-phosphate (Sigma–Aldrich, St. Louis, United States), non-essential amino acids, 100 units/mL penicillin, 100 μg/mL streptomycin (all except for mentioned, Gibco, Basel, Switzerland) and 0.1% Primocin (Invitrogen, San Diego, CA, United States). The medium was changed daily before and after loading and collected for further analysis. The osmolality of the three culture media containing different glucose concentrations was measured and is reported in the Supplementary Material (Supplementary Figure S1).

### 2.2 Metabolomics

For metabolite extraction, 10 µL of the conditioned media sample was transferred to an empty 2 mL Eppendorf sample tube and mixed with 80 µL of methanol and 10 µL of an isotopically labelled internal standard. Each sample was vortexed for 15 s and then centrifuged for 10 min at 10,000 RPM. The supernatant was transferred into an HPLC glass vial.

Targeted quantitative metabolite analysis was conducted using HILIC-based liquid chromatography and mass spectrometric detection. Metabolites were separated on ACQUITY Premiere BEH Z-HILIC 1.7 μm 2.1 × 100 mm analytical columns (Waters) utilizing a gradient elution method with 0.15% formic acid and 10 mM ammonium formate in water as mobile phase A and a solution of 0.15% formic acid and 10 mM ammonium formate in 85% acetonitrile as mobile phase B with the total analysis time of 18 min. The mobile phase flow rate was set at 0.4 mL/min, the injection volume at 2 μL, and the column temperature at 40 °C. For MS detection, an Orbitrap Exploris 120 mass spectrometer (Thermo Fisher Scientific) was used. The MS analysis was performed in ESI positive and ESI negative modes using full scan detection. The scan range was set from 50 to 600 m/z, and the mass resolution was set to 60,000. The ESI spray voltage was set to 3.5 kV in positive mode and 2.5 kV in negative mode; the gas heater temperature was set to 400 °C; the capillary temperature was set to 350 °C; the auxiliary gas flow rate was set to 12 arbitrary units; and the nebulizing gas flow rate was set to 50 arbitrary units. For quantitative analysis, seven-point calibration curves with internal standardization were used. TraceFinder 5.1 General Quan (Thermo Fisher Scientific) software was used for LC-MS data processing and quantification. Every reported metabolite was identified at level A using an authentic standard compound that had been previously mapped to the analytical system. Fresh unconditioned culture medium corresponding to each glucose concentration was analyzed in parallel and used as a reference, allowing normalization or subtraction of metabolites already present in the medium.

### 2.3 Histology

After 7 days of culture, endplates were removed from one side and whole IVDs were snap frozen in Tissue Freezing Medium (Leica Biosystems, Nussloch, Germany). Day 0 samples were equally processed directly after dissection. Transverse sections (10 µm) were obtained using a cryostat (Microm, Dreieich, Germany). Sections obtained were used to assess cell viability through lactate dehydrogenase/ethidium homodimer-1 (LDH/ETH1) staining (Stoddart, 2011; Secerovic et al., 2022). The slides were imaged with a ZEISS Axioscan 7, and the cell viability was calculated by adapting a protocol employed previously (Soubrier et al., 2025). Two 5.76 mm^2^ regions of interest (ROIs) were taken per region (outer AF, inner AF and NP). Cells stained in blue (LDH positive) and in blue/red (LDH and ETH1 positive) were counted as alive, while cells stained only in red (ETH1 positive) were counted as dead. The final viability was calculated for each region using a custom ImageJ workflow (Soubrier et al., 2025). Remaining histological sections were used for Safranin O/Fast Green staining (Supplementary Figure S2).

### 2.4 Disc height

Disc height was measured using a calliper immediately after dissection (day 0) and at day 7, following overnight free-swelling culture and following subsequent loading. Average height values were utilized to calculate the change in disc height, normalized to the height taken at day 0 after dissection.

### 2.5 Glycosaminoglycan release

The amount of sulphated glycosaminoglycans (sGAG) released into the culture medium was quantified by performing a 1.9-dimethylmethylene blue (DMMB) assay (Farndale et al., 1986) at pH 3 with chondroitin sulphate from bovine trachea (Sigma Aldrich) used as a standard and measuring absorbance at 530 nm. sGAG concentrations were calculated from the standard curve. Aliquots of the culture medium collected at each medium change were analyzed, and the daily sGAG values were added to obtain the cumulative sGAG concentration for each condition.

### 2.6 Gene expression

Tissue samples (150 mg) from the NP and AF were prepared for RNA isolation and gene expression analysis (Caprez et al., 2018). First, the samples were cut into small pieces, digested with 2 mg/mL pronase (Roche, Mannheim, Germany) for 1 h under the same glucose and oxygen conditions used during culture. Following digestion, samples were frozen in liquid nitrogen and pulverized. The resulting powdered tissue was carefully collected and added to TRI reagent, with the addition of 15 μL of polyacryl carrier (Molecular Research Center, Cincinnati, OH, United States), with the volume of TRI reagent adjusted based on the original weight of the NP tissue (ratio 10:1) to ensure an ample supply for RNA extraction. The samples were then promptly homogenized using a tissue lyser (Retsch GmbH and Co., Haan, Germany). After centrifugation, the lysate was collected, and phase separation was carried out by adding 100 μL of bromochloropropane for every 1 mL of TRI reagent, followed by centrifugation for phase separation. The aqueous phase was mixed with 70% ethanol, and the subsequent steps were performed using the QIAGEN RNeasy MINI kit according to the manufacturer’s protocol. For cDNA synthesis, the SuperScript VILO cDNA Synthesis Kit (Invitrogen) was employed, using 400 ng of RNA per sample. Quantitative real-time polymerase chain reaction (qRT-PCR) was executed on a QuantStudio 7 Flex PCR System (Applied Biosystems). The primers and probes used in the qRT-PCR are detailed in Supplementary Table S1. All data were analysed using the 2^−ΔΔCT^ method, with RPLP0 serving as the endogenous control.

### 2.7 Statistics

Statistical analysis was performed using GraphPad Prism 10 (version 10.1.2, GraphPad Software, LLC, San Diego, CA, United States) and R (version 4.3.1, Posit Software, Boston, MA, United States). Metabolomics data analysis was performed using MetaboAnalyst 5.0. All data are expressed as mean ± standard deviation and individual datapoints are shown. Non-parametric statistical tests were used as follows: For comparisons involving a single factor with independent groups, non-parametric Kruskal–Wallis tests, followed by Dunn’s *post hoc* test was employed. When datasets included repeated measures (comparison over days), a Mixed-Effects model was employed, followed by Tukey’s *post hoc* test for multiple comparisons. To assess overall sex differences, an Aligned Rank Transform (ART) ANOVA was performed, and where the main effect “sex” showed significance this was annotated. Comparisons between two groups (e.g., age of male vs. female donors) were made using an unpaired two-tailed Student’s t-test. A p-value <0.05 was considered statistically significant.

## 3 Results

### 3.1 Donor characteristics

To mitigate potential confounding factors arising from donor variability, we compared age and disc dimensions between male and female calves. The mean age of the calves was 8.81 ± 2.95 months, with no statistically significant difference (p = 0.73854) observed between the males (9.16 ± 2.56 months) and females (8.52 ± 3.45 months). Similarly, baseline measurements of disc height (M = 10.96 ± 1.58 mm; F = 11.94 ± 1.91 mm; p = 0.1224) and diameter (M = 16.78 ± 1.65 mm; F = 17.60 ± 1.85 mm; p = 0.1943) did not reveal any significant sex-related disparities. These findings confirm the comparability of male and female donor samples at baseline, indicating that the observed effects are not attributable to variations in age or disc size.

### 3.2 Metabolomics

**FIGURE 1:**
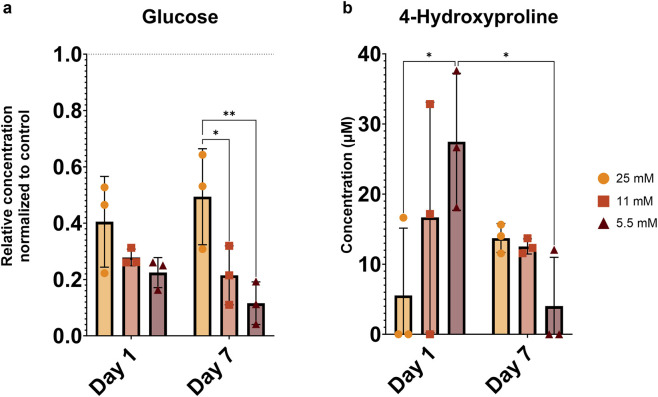
**(a)** Glucose and **(b)** 4-hydroxyproline concentrations measured at day 1 and day 7 in media collected after overnight culture. Glucose concentrations were normalized to the corresponding fresh culture medium with known glucose content. Data are expressed as mean ± SD (n = 3; 3 M). Significant differences are indicated by black asterisks (**p ≤ 0.01, *p ≤ 0.05).

**FIGURE 2:**
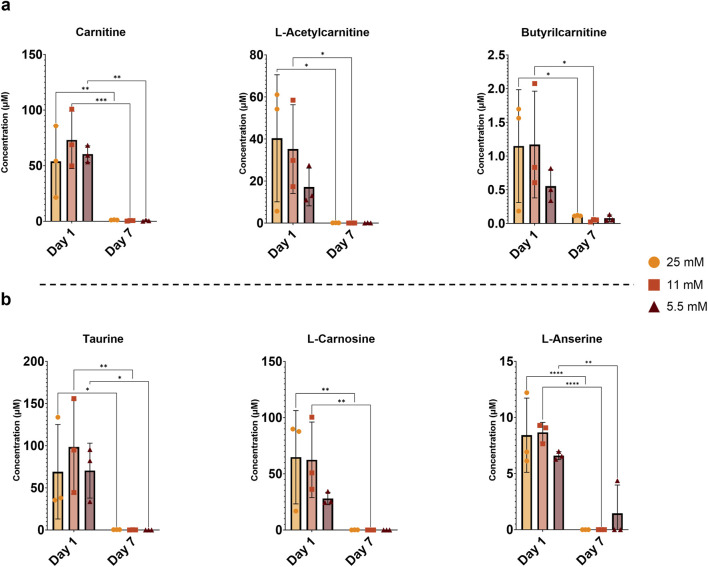
**(a)** β-oxidation-related and **(b)** antioxidant and osmoregulatory metabolites measured at day 1 and day 7 in media collected after overnight culture. Data are expressed as mean ± SD (n = 3; 3 M). Significant differences are indicated by black asterisks (****p ≤ 0.0001, ***p ≤ 0.0001, **p ≤ 0.01, *p ≤ 0.05).

To investigate glucose levels and the release of metabolites in the medium, metabolomics analysis was conducted on conditioned media of day 1 and day 7. As expected, the relative concentration of glucose (Figure 1a) was significantly higher in the 25 mM group compared to the other groups in the overnight medium on day 7. Levels of 4-hydroxyproline (Figure 1b), which indicate collagen breakdown, showed a transient release pattern across all conditions. On day 1, the 5.5 mM group exhibited higher levels of 4-hydroxyproline than both the 25 mM group and its equivalent group at day 7. Markers of fatty acid β-oxidation, including free carnitine, L-acetylcarnitine, and butyrylcarnitine, generally decreased from day 1 to day 7 across all glucose groups (Figure 2a), reaching statistical significance for free carnitine at all glucose concentrations. Additionally, the levels of metabolites known to have a role in antioxidant protection and osmoregulation, such as taurine, L-carnosine, and L-anserine, decreased from day 1 to day 7 across all glucose conditions (Figure 2b), with statistically significant reductions observed for taurine and L-anserine at all concentrations, while L-carnosine did not reach significance at the lowest glucose level. Due to the limited number of donor samples (n = 3) for this analysis, a comprehensive examination of sex-specific differences was not feasible. A complete list of all measured metabolites is available in the Supplementary Material (Supplementary Table S2). Together, these findings indicate time-dependent changes consistent with a metabolic adaptation to the *ex vivo* culture environment, characterized by reduced β-oxidation activity and diminished availability of metabolites involved in antioxidant protection and osmoregulation over time.

### 3.3 Cell viability

**FIGURE 3:**
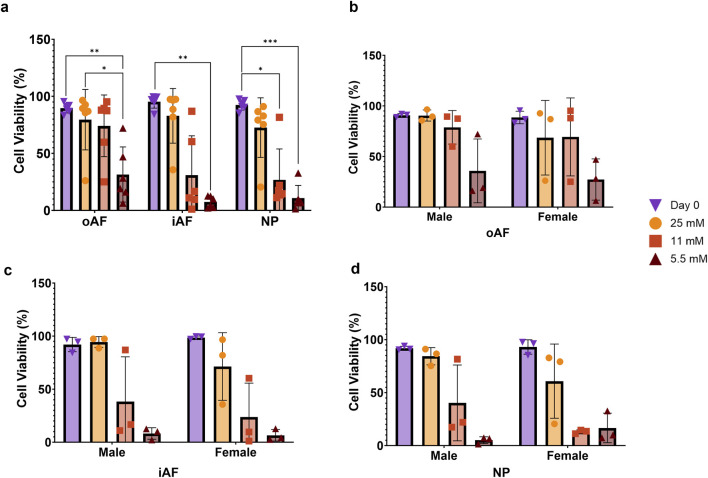
Cell viability in the outer annulus fibrosus (oAF), inner annulus fibrosus (iAF), and nucleus pulposus (NP) at different glucose concentrations (25 mM, 11 mM, and 5.5 mM) after 1 week of physiological loading (2 h/day). **(a)** Shows the full dataset (n = 6), while **(b–d)** present data from male and female donors separately for the oAF, iAF, and NP, respectively. Data are expressed as mean ± SD (n = 6; 3 M/3 F). Significant differences are indicated by black asterisks (***p ≤ 0.001, **p ≤ 0.01, *p ≤ 0.05).

After 7 days of culture in the presence of varying concentrations of glucose, we assessed cell viability using LDH/ETH1 (Supplementary Figure S3). The results presented in Figure 3a show the impact of glucose deprivation on cell viability. In samples cultured for 1 week with 2 h of physiological loading per day, cell viability remained consistently high (79.5%, 82.9%, and 72.6%) in the oAF, iAF and NP when exposed to 25 mM of glucose, showing no significant difference compared to the Day 0 sample (89.62%, 95.2%, and 92.5%). However, at lower glucose concentrations, while the oAF seemed to be unaffected at 11 mM, there was a dramatic drop in all the other zones, reaching values below or around 10% at 5.5 mM in the NP. Data were further analysed to reveal also a potential difference in viability between male and female donors respectively in oAF, iAF, and NP (Figures 3b–d). No significant differences were observed between male and female donors at the same concentration of glucose. These results indicate that whole-disc cell viability is strongly dependent on extracellular glucose availability, with the inner AF and NP being particularly vulnerable to glucose reduction under the present culture conditions.

### 3.4 Disc height change

**FIGURE 4:**
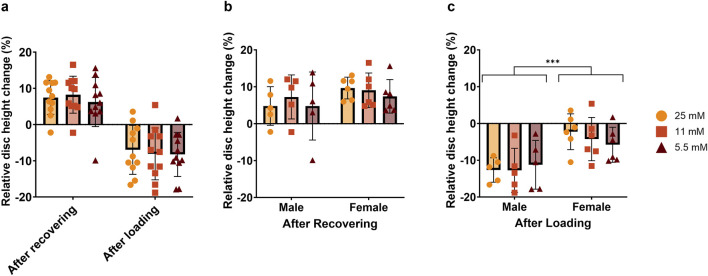
Relative height changes at day 7 after overnight recovery and after loading. **(a)** Shows the full dataset, while **(b,c)** show male and female donors separately. Data are expressed as mean ± SD (n = 11; 5 M/6 F). Significant differences are indicated by black asterisks (***p ≤ 0.001).

After 1 week, we measured relative disc height changes following overnight free-swelling culture and after 2 h of loading. Although no significant differences were observed overall among the glucose groups (Figures 4a,b), there was a greater height loss after loading in male compared to female IVDs across all the groups (Figure 4c). This pattern suggests that glucose concentration has a limited effect on short-term disc height maintenance, while sex-dependent differences may influence the mechanical response to loading.

### 3.5 Cumulative sGAG release

**FIGURE 5:**
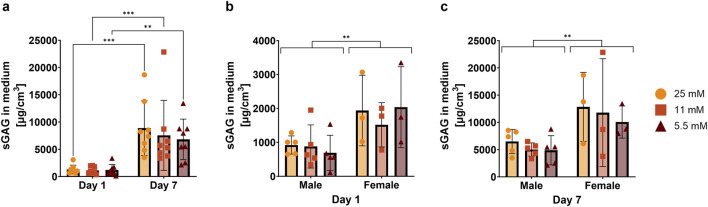
Cumulative release of sulfated glycosaminoglycans (sGAGs) into the culture medium. **(a)** Shows the full dataset, while **(b,c)** show male and female donors separately at day 1 and day 7. Data are expressed as mean ± SD (n = 8; 5 M/3 F). Significant differences are indicated by black asterisks (***p ≤ 0.001, **p ≤ 0.01, *p ≤ 0.05).

We measured cumulative sGAG release in culture medium from day 1 to day 7 as a sensitive indicator of matrix turnover and tissue response to the culture conditions, while preserving tissue integrity for complementary analyses. Cumulative release was calculated from daily medium collections, whereas values at day 1 and day 7 are shown for clarity. As expected for cumulative measurements, sGAG release increased significantly over time, while no significant differences were observed between glucose groups at the same time points (Figure 5a). Interestingly, at both day 1 and day 7, cumulative sGAG release was consistently higher in female donors compared to male donors (Figures 5b,c). Overall, these data suggest potential sex-related differences in matrix turnover during culture, despite the absence of significant glucose-dependent effects on cumulative sGAG release.

### 3.6 Gene expression analysis of anabolic and catabolic markers

**FIGURE 6:**
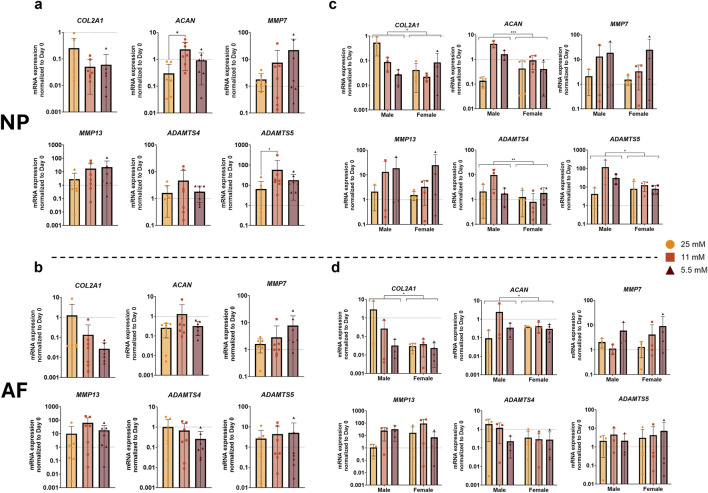
Gene expression of catabolic and anabolic markers in NP **(a)** and AF **(b)** cells after 1 week of physiological loading, normalized to the day 0 control. **(c,d)** Show male and female donors separately in NP and AF cells, respectively. Data are expressed as mean ± SD (n = 7; 3 M/4 F). Significant differences are indicated by black asterisks (, ***p ≤ 0.001, **p ≤ 0.01, *p ≤ 0.05).

We conducted a gene expression analysis to evaluate the impact of varying glucose concentrations on anabolic and catabolic marker genes after 7 days. For most genes, we observed no significant differences between the groups. In the NP (Figure 6a), *ACAN* expression was significantly increased in the 11 mM group compared to the 25 mM group. Generally, the regulation of matrix metalloproteinases (MMPs) was similar between AF (Figure 6b) and NP, and no distinct trends were observed for *ADAMTS4*, while *ADAMTS5* was significantly higher in the 11 mM group compared to the 25 mM group in the NP.

When examining sex differences, significant variations were observed in the expression of matrix-related genes between male and female donors. *COL2A1* and *ACAN* expression differed significantly between sexes in both the NP and AF (Figures 6c,d), with male donors generally exhibiting higher expression levels than females. In the NP, sex-dependent differences were also detected for catabolic markers, with both *ADAMTS4* and *ADAMTS5* showing significantly higher expression in male compared to female samples. Collectively, these results indicate that glucose availability modulates matrix-related gene expression in a sex-dependent manner, particularly within the NP.

### 3.7 Gene expression of glucose transporter and apoptosis markers

**FIGURE 7:**
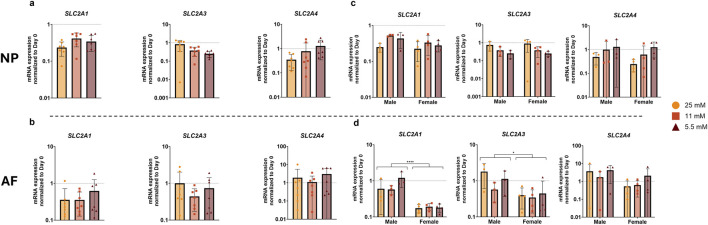
Gene expression of glucose transporters in **(a)** NP and **(b)** AF tissue after 1 week of physiological loading, normalized to the day 0 control. **(c,d)** Show male and female donors separately in NP and AF cells, respectively. Data are expressed as mean ± SD (n = 7; 3 M/4 F). Significant differences are indicated by black asterisks (****p ≤ 0.0001, *p ≤ 0.05).

We conducted a gene expression analysis to evaluate the effects of varying glucose concentrations on glucose transporters and apoptosis markers. The analysis of glucose transporters revealed no significant differences between glucose concentration groups in either the NP or AF (Figures 7a,b).

When examining sex differences, there were no significant differences in the NP (Figure 7c). In contrast, sex-dependent differences were observed in the AF, where both *SLC2A1* and *SLC2A3* expression levels were significantly higher in male compared to female donors (Figure 7d).

**FIGURE 8:**
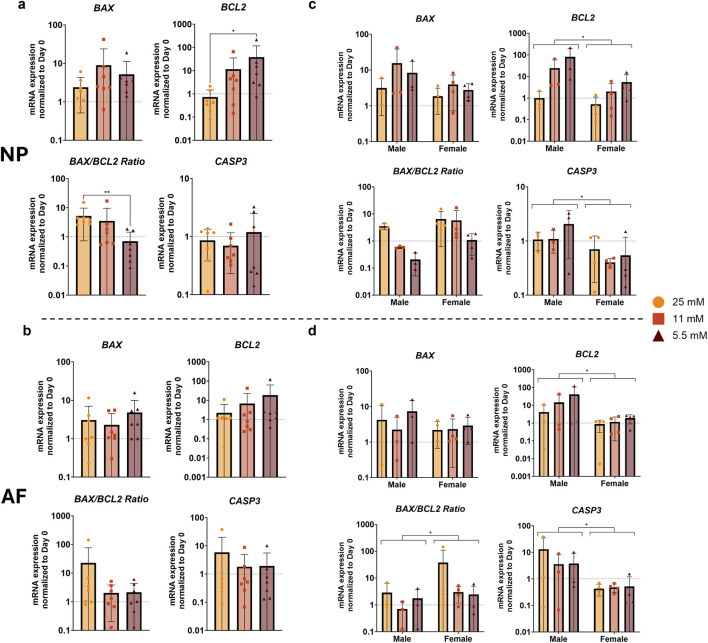
Gene expression of apoptotic markers in **(a)** NP and **(b)** AF tissue after 1 week of physiological loading, normalized to the day 0 control. **(c,d)** Show male and female donors separately in NP and AF cells, respectively. Data are expressed as mean ± SD (n = 7; 3 M/4 F). Significant differences are indicated by black asterisks (**p ≤ 0.01, *p ≤ 0.05).

In our examination of apoptosis markers, we observed limited glucose-dependent effects. In the NP, *BCL2* expression was significantly higher in the 5.5 mM group compared to the 25 mM group, whereas the *BAX/BCL2* ratio showed the opposite pattern, being significantly higher at 25 mM compared to 5.5 mM glucose (Figure 8a). No significant glucose-dependent differences were observed for apoptosis markers in the AF (Figure 8b). When considering sex differences, both *BCL2* and *CASP3* were significantly upregulated at the lowest glucose concentration in males compared to females in the NP and AF (Figures 8c,d). In addition, the *BAX/BCL2* ratio was significantly lower in males than in females in the AF, while no significant sex-related difference was detected in the NP. Together, these findings suggest that glucose transporters and apoptotic markers are modestly influenced by glucose availability but show pronounced sex-dependent differences across disc regions.

## 4 Discussion

This study investigated the impact of varying glucose concentrations on whole bovine IVDs culture subjected to mechanical loading. While disc cells are known to thrive in harsh environments with low glucose, oxygen, and pH (Shan et al., 2019), our findings suggest that in an organ culture setting glucose deprivation can compromise cell viability and alter gene expression.

Recent studies have suggested a significant correlation between diabetes mellitus (DM) and IDD (Cannata et al., 2020). Although clinical evidence is inconsistent, laboratory studies strongly implicate DM as a contributing factor to IDD (Alpantaki et al., 2019). In diabetic conditions, hyperglycemia leads to cell senescence, apoptosis, and ECM catabolism in IVDs (Chen et al., 2022; Lintz et al., 2022). Multiple mechanisms, including microangiopathy, cellular senescence, and advanced glycation end products, have been proposed to contribute to IDD in diabetic patients (Li et al., 2024). Notably, diabetic hyperglycemia is characterized by blood glucose levels ranging from approximately ≥11 mM (American Diabetes Association Professional Practice Committee, 2024). While these concentrations exceed physiological IVD glucose levels, they are substantially lower than the 25 mM glucose frequently employed in many *ex vivo* IVD culture systems (McDonnell and Buckley, 2021). Determining the lowest glucose concentration that still supports cell viability is therefore critical for developing more physiologically relevant models.

Nonetheless, we observed that cell viability was maintained at the glucose concentration of 25 mM, but significantly declined at lower levels, particularly in the iAF and NP regions. This aligns with prior studies demonstrating that NP cells are more sensitive to glucose deprivation than AF cells (Rinkler et al., 2010). This differential sensitivity may be due to the greater metabolic demands of NP cells, which rely more heavily on anaerobic glycolysis for ATP production (Rinkler et al., 2010) as well as the nutrient diffusion gradients within the IVD. This gradient is dependent on the region and porosity in the CEP (Wu et al., 2016), and it also varies with disc mid-height and tissue health (Workineh et al., 2025). Notably, NP explants cultured without the CEP can remain viable even at physiological glucose levels (2 mM), emphasizing the critical role of CEP permeability in nutrient supply (Padrona et al., 2024). While the IVDs were subjected to dynamic loading, physiological loading primarily induces only limited fluid convection, thereby ensuring that solute transport remains predominantly governed by diffusion (Salzer et al., 2025). Consequently, the intrinsic CEP-dependent gradient persists even under loaded conditions, suggesting that improving CEP permeability or introducing enhanced perfusion could potentially help to overcome this limitation in *ex vivo* systems.

Although glucose consumption by the discs was not directly quantified, the residual glucose levels measured in the conditioned media indicate concentration-dependent differences in glucose utilization. Glucose uptake by cells is mediated by specific transporters and is strongly influenced by extracellular glucose availability (Lienhard et al., 1992). In the present study, we observed that substantial glucose remained in the medium at 25 mM, compared to 11 mM and 5.5 mM groups. Since NP cells rely predominantly on glycolysis for ATP production (Bibby et al., 2005), the pronounced loss of viability observed at 11 mM and 5.5 mM may primarily reflect insufficient glucose supply rather than altered cellular demand alone, especially in regions distant from the nutrient source. The culture and loading process can independently disrupt the equilibrium of the ECM, as IVDs adapt from their native *in vivo* environment to *ex vivo* conditions, and this can lead to alterations in cell phenotype and the expression of genes involved in matrix synthesis and degradation (Zhang et al., 2025). This is particularly evident for short-duration culturing when the cells have little time to adapt to the environmental changes. This observation is supported by our metabolomics data, which indicated a higher release of taurine, L-carnosine, and L-anserine at day 1 compared to day 7. These metabolites are known to counteract oxidative and osmotic stress through several mechanisms. Notably, taurine can reduce oxidative stress in rat IVDs (Lin et al., 2024), and it has also been detected in healthy but not in degenerated human discs (Pacholczyk-Sienicka et al., 2015). Additionally, we noted a transient increase in 4-hydroxyproline, a marker of collagen breakdown, at day 1 relative to day 7, indicating early matrix remodeling during this adaptation phase.

The impaired β-oxidation observed by the reduction in metabolites from day 1 to day 7 in this pathway suggests mitochondrial dysfunction, a common phenomenon in degenerative diseases. Both IVD and articular cartilage degeneration are associated with impaired β-oxidation and mitochondrial dysfunction, which are linked to oxidative stress, limited oxygen, and metabolic imbalances that reduce ATP production and degrade the matrix (Zhu et al., 2022; Song et al., 2024; Tan et al., 2025). This metabolic response might be an early adaptation to the altered *ex vivo* environment. Optimizing bioreactor culture conditions, such as introducing gradual adaptation periods, or nutrient preconditioning, could help reduce this stress and better maintain disc cell homeostasis.

Despite the different glucose availability between the groups, the glucose transporters did not show clear glucose-driven regulation. Nonetheless, SCL2A1 and SCL2A3 were higher in males than in females, which may imply a possible sex-specific regulation of glucose uptake.

Interestingly, the anti-apoptotic marker BCL2 was inversely related to glucose concentration, suggesting that high glucose levels may also negatively impact cell viability, possibly due to osmotic stress or metabolic disturbances. The upregulation of both BCL2 and CASP3 in males compared to females in the NP suggests sex-specific responses to glucose stress.

Male donors exhibited higher expression of several markers than female donors. This pattern may reflect a stronger metabolic or adaptive response in male discs under changing nutrient conditions. This aligns with recent findings of sex-based differences in biomechanics, biochemistry, and inflammatory responses to injury in rats (Kenawy et al., 2023), as well as higher proteoglycan levels observed in the lumbar discs of men compared to women (Bonnheim et al., 2023). Overall, our findings highlight possible sex-dependent adaptations to glucose availability, underscoring the need for further studies to elucidate the mechanisms underlying these differences.

This study has some limitations. First, the use of bovine rather than human discs, despite their similarities, may limit the translatability of our findings (Tang et al., 2022). Additionally, the discs were sourced from young animals which, at this age, may not yet have reached full skeletal and sexual maturity. Hormonal influences could therefore be less pronounced than in adult animals, and it is possible that sex-related differences in glucose responses would be even more evident in older donors. Additionally, the limited number of donors included in the metabolomics analysis may not have fully accounted for inherent variability between samples and did not allow for sex-based comparison. Therefore, these data are presented as exploratory and hypothesis-generating rather than conclusive. Furthermore, this study only examined static culture and uniaxial loading, whereas a more complex culture and loading regime may be necessary to better replicate *in vivo* conditions and reflect the physiological environment. Finally, in this study, we focused on a well-established panel of anabolic and catabolic markers, complemented by glucose transporters and apoptosis-related genes, to capture key aspects of disc cell responses to altered glucose availability. While this targeted approach provides relevant biological insight, the analysis of additional metabolism-related genes, such as those involved in glycolysis, mitochondrial function, or fatty acid metabolism, would further strengthen the interpretation of glucose utilization and metabolic adaptation during organ culture. Future studies incorporating such markers, ideally alongside direct measurements of glucose consumption, will be important to further refine metabolically relevant *ex vivo* IVD models. In addition, protein-level validation of intracellular markers was not performed. In ECM-rich IVD, protein extraction and detection of low-abundance, non-secreted proteins remain technically challenging. Additionally, the relatively short culture duration (1 week) is expected to capture early transcriptional adaptations rather than structural changes in ECM composition. Moreover, investigation of the specific types of cell death occurring under glucose deprivation will be required to provide mechanistic insights into this process.

Despite these limitations, this study provides insights into the complex interplay between glucose availability under a physiological loading regime, and the metabolic and catabolic responses of IVD cells. While a physiological level of glucose appears to maintain cell viability and matrix homeostasis, both glucose deprivation and excess can potentially contribute to IDD through different mechanisms. A better understanding of these processes may inform the development of targeted therapeutic strategies to address IVD pathologies.

## 5 Conclusion

In this study, we investigated the effects of varying glucose concentrations on the metabolic activity and gene expression of IVD cells under mechanical loading.

Our results demonstrate the following.

1. Cell viability in a bovine *ex vivo* model can be maintained only with a glucose concentration of 25 mM. Lower glucose levels, particularly 5.5 mM, significantly reduce cell viability in both the NP and inner AF.
2. Metabolomic data showed early changes in metabolites related to β-oxidation, antioxidant defense, and osmotic regulation, indicating an acute metabolic stress response. IVD isolation itself may induce an initial stress effect that impacts the cells.
3. Some investigated markers showed sex-dependent regulation, indicating that male and female discs may respond differently to glucose availability, even though viability was not affected by sex.

*Ex vivo* models play an important role in the development and testing of new therapies. However, conditions that differ from physiological surroundings may limit the translation of new findings. Therefore, refining *ex vivo* models is essential to enhance the relevance and predictability of these studies. Glucose deprivation compromises the survival of cells in the inner part of the disc; hence, low glucose medium can be utilized to simulate degenerative conditions, potentially in combination with inflammatory cytokines. Future investigations are vital for improving and validating organ models, making them effective tools for preclinical IVD and spine research.

## Data Availability

The original contributions presented in the study are included in the article/Supplementary Material, further inquiries can be directed to the corresponding author.
