# Supplementary material for: Glucose concentration modulates bovine intervertebral disc response under physiological loading
Source: Front Bioeng Biotechnol. 2026 Mar 26;14:1751565. doi: 10.3389/fbioe.2026.1751565 (PMC13061861; doi:10.3389/fbioe.2026.1751565)
Supplement: Supplementary file 1 [file Supplementaryfile1.docx]

Supplementary Material

**Table S1.** List and details of primers and probes used for gene expression analysis

Table S1 List and details of primers and probes used for gene expression analysis

| **Gene** | | **Assay Details** | |
| --- | --- | --- | --- |
| *RPLP0* | Ribosomal Protein Lateral Stalk Subunit P0 | ThermoFisher | Bt03218086_m1 |
| *ACAN* | Aggrecan | Primer forward (5′-3′) | 5'-CCA ACG AAA CCT ATG ACG TGT ACT-3' |
|  |  | Primer reverse (5′-3′) | 5'-GCA CTC GTT GGC TGC CTC-3' |
|  |  | Probe (5′FAM/3′TAMRA) | 5'-ATG TTG CAT AGA AGA CCT CGC CCT CCA T-3' |
| *ADAMTS4* | ADAM metallopeptidase with thrombospondin type 1 motif 4 | Primer forward (5′-3′) | 5'-CCC CAT GTG CAA CGT CAA G-3' |
|  |  | Primer reverse (5′-3′) | 5'-AGT CTC CAC AAA TCT GCT CAG TGA-3' |
|  |  | Probe (5′FAM/3′TAMRA) | 5'-AGC CCC CGA AGG GCT AAG CGC-3' |
| *ADAMTS5* | ADAM Metallopeptidase with Thrombospondin Type 1 Motif 5 | Primer forward (5′-3′) | 5'-GAT GGT CAC GGT AAC TGT TTG CT-3' |
|  |  | Primer reverse (5′-3′) | 5'-GCC GGG ACA CAC CGA GTA C-3' |
|  |  | Probe (5′FAM/3′TAMRA) | 5'-AGG CCA GAC CTA CGA TGC CAG CC-3' |
| *BAX* | BCL2 associated X, apoptosis regulator | ThermoFisher | Bt03211776_m1 |
| *BCL2* | BCL2 Apoptosis Regulator | ThermoFisher | Bt04298952_m1 |
| *CASP3* | Caspase 3 | ThermoFisher | Bt03250956_g1 |
| *COL2A1* | Collagen Type II Alpha 1 Chain | Primer forward (5′-3′) | 5'-AAG AAA CAC ATC TGG TTT GGA GAA A-3' |
|  |  | Primer reverse (5′-3′) | 5'-TGG GAG CCA GGT TGT CAT C-3' |
|  |  | Probe (5′FAM/3′TAMRA) | 5'-CAA CGG TGG CTT CCA CTT CAG CTA TGG-3' |
| *MMP7* | Matrix Metallopeptidase 7 (Matrilysin) | Primer forward (5′-3′) | 5'-GTG GCC AAG GCC TTC AAA-3' |
|  |  | Primer reverse (5′-3′) | 5'-CTT CTT GCA AAG CCA ATC ATG A-3' |
|  |  | Probe (5′FAM/3′TAMRA) | 5'-AGC GAA GCA ATC CCA CTG ACG TTT AAG A-3' |
| *MMP13* | Matrix Metallopeptidase 13 (Collagenase 3) | Primer forward (5′-3′) | 5'-CCA TCT ACA CCT ACA CTG GCA AAA G-3' |
|  |  | Primer reverse (5′-3′) | 5'-GTC TGG CGT TTT GGG ATG TT-3' |
|  |  | Probe (5′FAM/3′TAMRA) | 5'-TCT CTC TAT GGT CCA GGA GAT GAA GAC CCC-3' |
| *SLC2A1* | Solute Carrier Family 2 Member 1 (GLUT1) | ThermoFisher | Bt03215313_m1 |
| *SLC2A3* | Solute Carrier Family 2 Member 3 (GLUT3) | ThermoFisher | Bt03259519_gH |
| *SLC2A4* | Solute Carrier Family 2 Member 4 (GLUT4) | ThermoFisher | Bt03215316_m1 |


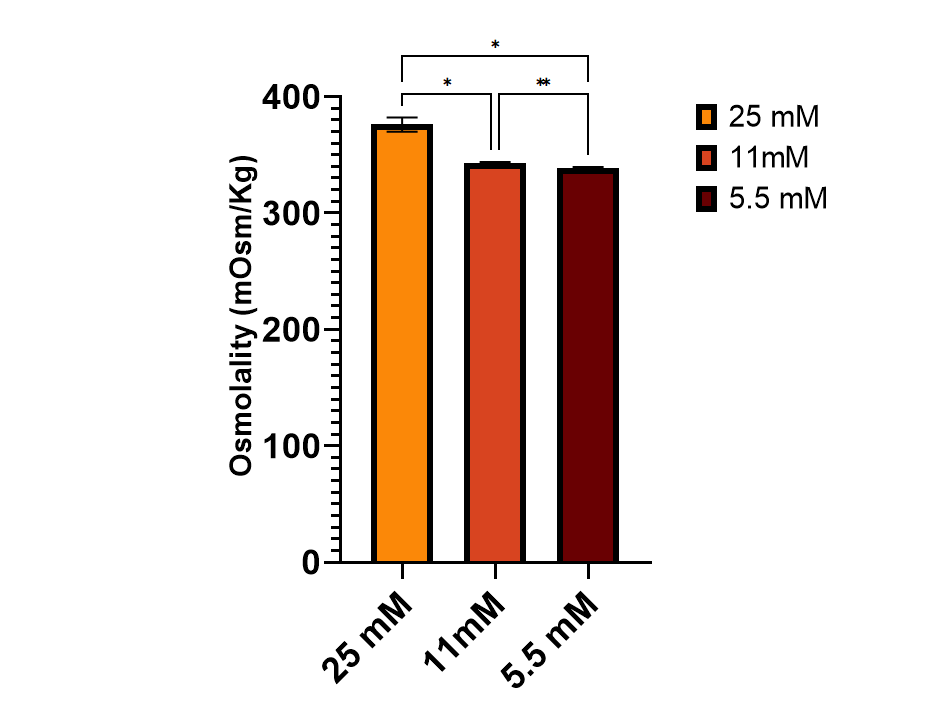


**Figure S1 Osmolality of culture media containing different glucose concentrations**. Osmolality was measured in technical triplicates for media containing 5.5, 11, or 25 mM glucose using a freezing-point osmometer. Values increased significantly with glucose concentration (repeated-measures one-way ANOVA, p < 0.05). Tukey’s post hoc test showed significant differences between all conditions (p < 0.05, *p < 0.01). Bars represent mean ± SD of three measurements.

Table S2 List of measured metabolites

| 4-Hydroxyproline | Aminoadiphic Acid | Aminoisobutyric Acid | Amino-n-butyric Acid | Butyrylcarnitine |
| --- | --- | --- | --- | --- |
| Carnitine | Citrulline | Creatinine | Ethanolamine | Glycine |
| Hydroxylysine | Isovalerylcarnitine | Kynurenine | L-Acetylcarnitine | L-Alanine |
| L-Anserine | L-Arginine | L-Asparagine | L-Aspartic Acid | L-Carnosine |
| L-Cystine | L-Glutamic Acid | L-Glutamine | L-Histidine | L-Isoleucine |
| L-Leucine | L-Lysine | L-Methionine | L-Phenylalanine | L-Proline |
| L-Sarcosine | L-Serine | L-Threonine | L-Tryptophan | L-Tyrosine |
| Propionylcarnitine | Serotonine | Taurine | Glucose |  |


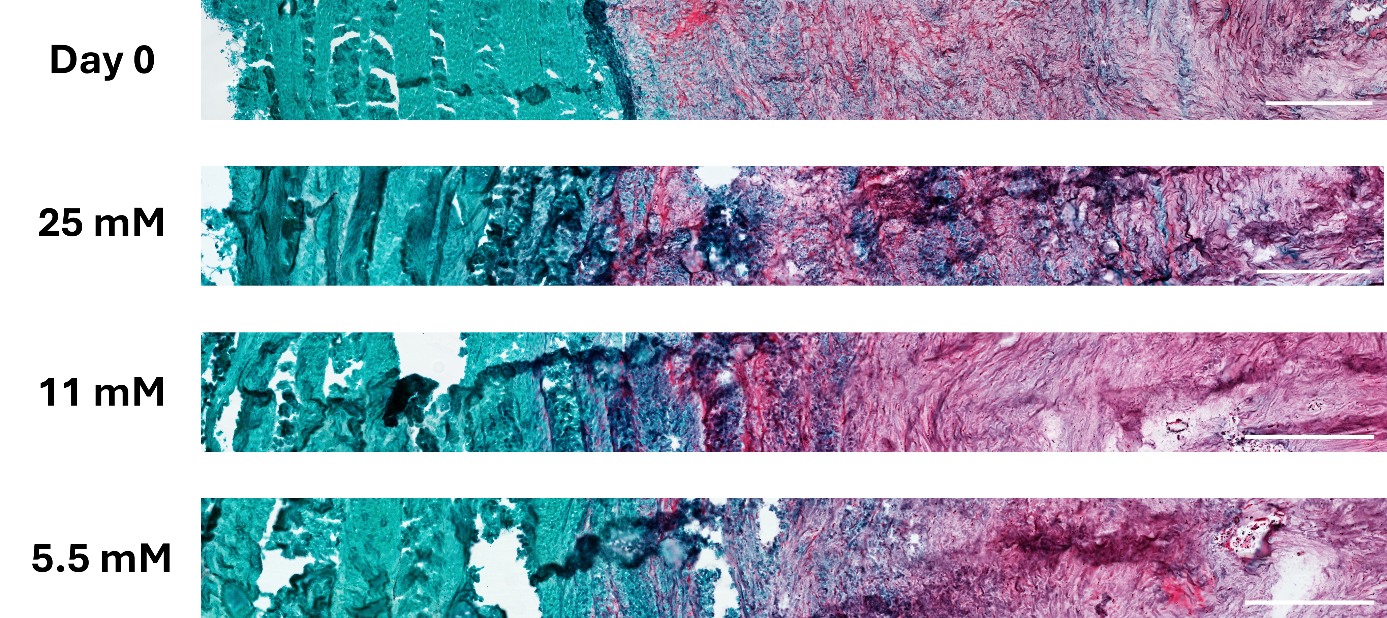


**Figure S2. Representative images of Safranin-O/Fast Green staining on transverse IVD section**. Sections were stained with 0.1% Safranin-O to visualize proteoglycans and 0.02% Fast Green to highlight collagen distribution. Cell nuclei were subsequently counterstained with Weigert’s Haematoxylin. Scale bar (bottom right in each picture) = 1 mm.


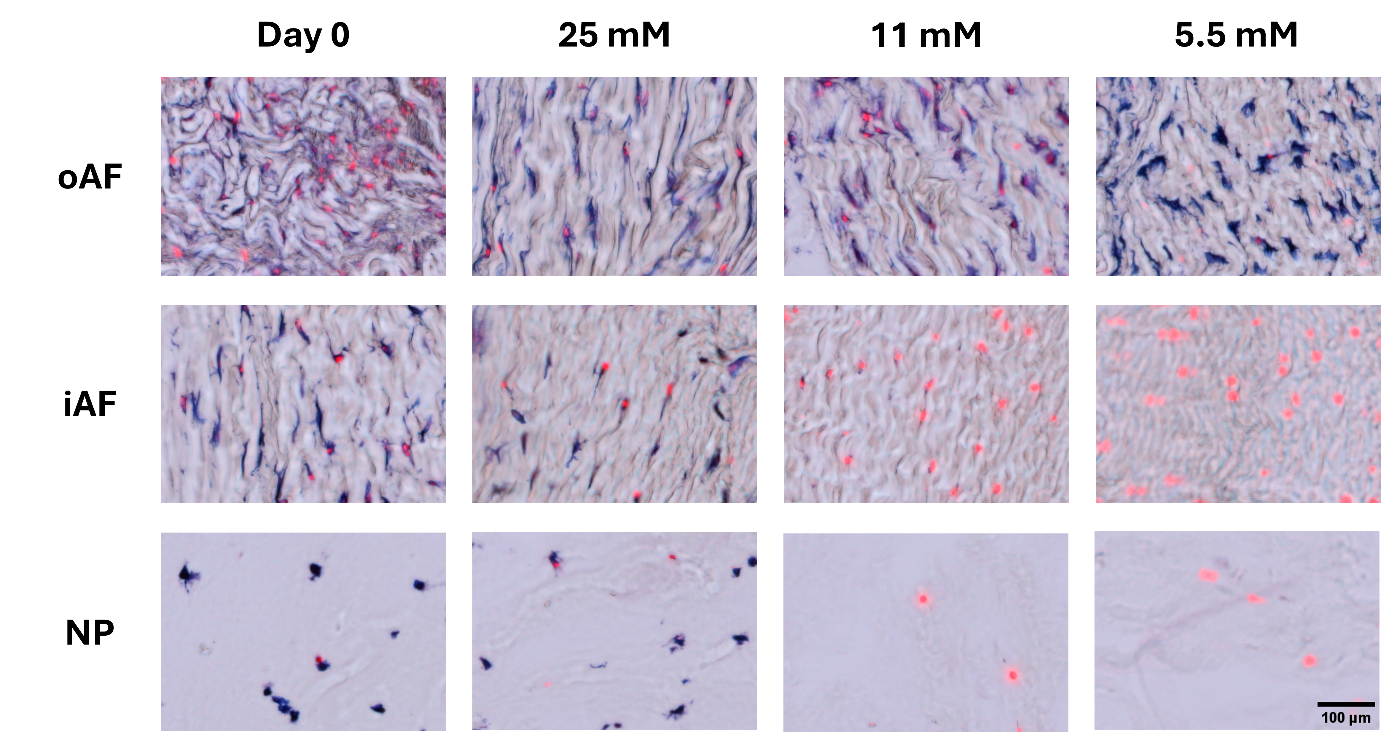


**Figure S3. Representative images of LDH / ETH1 staining used to assess cell viability.**
